# Srs11‐92, a ferrostatin‐1 analog, improves oxidative stress and neuroinflammation via Nrf2 signal following cerebral ischemia/reperfusion injury

**DOI:** 10.1111/cns.14130

**Published:** 2023-02-27

**Authors:** Yuhua Chen, Wei He, Hong Wei, Cuicui Chang, Lingjian Yang, Jiao Meng, Tianlin Long, Quanhua Xu, Chi Zhang

**Affiliations:** ^1^ Department of Central Laboratory Xi'an Peihua University Xi'an China; ^2^ Department of Neurosurgery Bijie Traditional Chinese Medicine Hospital Bijie China; ^3^ Department of Neurosurgery Qilu Hospital of Shandong University Qingdao China; ^4^ Department of Rehabilitation Teaching and Research Xi'an Siyuan University Xi'an China; ^5^ School of Chemistry & Chemical Engineering Ankang University Ankang China; ^6^ Department of Neurosurgery Xiangya Hospital, Central South University Changsha China; ^7^ The Institute of Skull Base Surgery and Neurooncology at Hunan Province Changsha China

**Keywords:** ferroptosis, ischemic stroke, neuroinflammation, oxidative stress, Srs11‐92

## Abstract

**Aim:**

Ferroptosis is increasingly becoming to be considered as an important mechanism of pathological cell death during stroke, and specific exogenous ferroptosis inhibitors have the ability to reverse cerebral ischemia/reperfusion injury. However, research on Srs11‐92 (AA9), a ferrostatin‐1 (Fer‐1) analog, in preclinical studies is limited.

**Methods:**

In the middle cerebral artery occlusion‐reperfusion (MCAO/R) mice model or oxygen–glucose deprivation/reperfusion (OGD/R) cell model, Fer‐1, AA9, and/or ML385 were administered, and brain infarct size, neurological deficits, neuronal damage, oxidative stress, and neuroinflammation were determined after the damage, in vitro and in vivo.

**Results:**

Fer‐1 and AA9 improved brain infarct size, neuronal damage, and neurological deficits in mice model of MCAO/R, and inhibited the overloaded iron deposition, ROS accumulation, and neuroinflammation response: it also increased the expression of GPx4, Nrf2, and HO‐1 and suppressed the expression of HMGB1 and NF‐κB p65 in the epicenter of injured hippocampal formation. However, Nrf2 inhibitor ML385 reversed the neuroprotective effect of AA9, including the oxidative stress and neuroinflammation. In vitro studies showed that AA9 relieved OGD/R‐induced neuronal oxidative stress and neuroinflammation via the Nrf2 pathway, which was impaired by ML385 in primary neurons.

**Conclusion:**

The findings imply that Fer‐1 analog AA9 may be suitable for further translational studies for the protection of neuronal damage via Nrf2 signal pathway‐mediated oxidative stress and neuroinflammation in stroke and others neurological diseases.

## Graphical abstract

Srs11‐92 (AA9), a Fer‐1 analog (ferrostatins), is an effective inhibitor of ferroptosis, which inhibits neuronal death, nerve injury, and motor dysfunction after ischemic stroke, via the regulation of Nrf2‐mediated oxidative stress and neuroinflammation following cerebral ischemia/reperfusion injury.

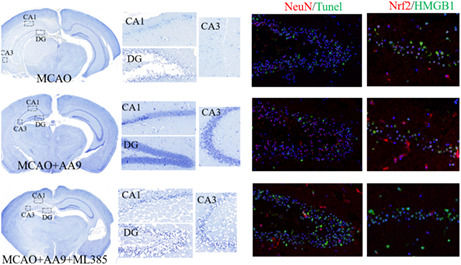

## 1 INTRODUCTION

Stroke is the second leading cause of death and disability worldwide.^1^ As the global population of people aged 65 years and above increases rapidly, so does the incidence of stroke, and the overall stroke burden has shifted to younger age groups.^1^, ^2^, ^3^ Stroke is mainly divided into two types, namely, ischemic stroke (about 80%–85%) and hemorrhagic stroke: ischemic stroke is caused by cerebral artery occlusion, while hemorrhagic stroke is caused by non‐traumatic vascular rupture in the brain parenchyma.^1^ Because of cerebral tissue ischemia or hemorrhage, the normal blood supply to neurons is disrupted, which further promotes a series of pathophysiological reactions leading to neuronal death. Also, the process involves a combination of many mechanisms, including neuronal overexcitation, mitochondrial death, free radical release, apoptosis, necrosis, autophagy, and neuroinflammation.^4^ Currently, intravenous thrombolytic therapy with recombinant tissue plasminogen activator is the only approved treatment for acute ischemic stroke; however, it also has significant clinical limitations and a narrow treatment window, which increases the risk of cerebral intracerebral hemorrhage (ICH).^5^ As a result, most patients are unable to fully recover and regain their normal neurological functions, which is the main reason for the burden on their families and society.^6^ So, developing new more effective approaches are of priority, to reduce the mortality and morbidity of ischemic stroke.

Studies have confirmed that neuronal death mainly includes apoptosis, necrosis, autophagy, pyroptosis, and so forth^7^; however, these mechanisms still cannot fully explain the early brain injury caused by acute central nervous system diseases. Ferroptosis, a non‐apoptotic form of programmed cell death (PCD), is characterized by the accumulation of intracellular lipid peroxides and related metabolites.^8^ Ferroptosis is morphologically, biochemically, and genetically different from other forms of PCD and is involved in a variety of diseases.^9^ In recent years, more and more studies have found that ferroptosis is increasingly being considered as an important mechanism of pathological cell death during stroke and other acute brain injury.^10^ Previous studies have shown that focal cerebral ischemia rapidly leads to increased free iron levels in ischemic tissue, as soon as 1 h.^11^ With the aid of T2*‐weighted magnetic resonance imaging, researchers have demonstrated hypothalamic signaling in almost all ipsilateral ischemic stroke patients, which manifests shortly after stroke onset and is related to iron deposition.^12^ In middle cerebral artery occlusion‐reperfusion (MCAO/R) model animals, iron intake is positively correlated to the infarct volume, and iron inhibitors have the effect of improving neurological impairment.^13^ Deferoxamine (DFO), as a high affinity iron chelator, has been approved by the USA Food and Drug Administration for the treatment of iron overload.^13^ Preclinical studies have shown that early intervention of ferroptosis inhibitor ferrostatin‐1 (Fer‐1) or liproxstatin‐1 effectively improves neuronal damage and functional defects in MCAO mice.^14^ It has been suggested that specific exogenous ferroptosis inhibitors may have the ability to reverse cerebral ischemia/reperfusion injury (IRI).^15^

The specific ferroptosis inhibitors Liproxstatin‐1 and Ferrostatin‐1 can effectively prevent ischemia–reperfusion injury in a dose‐dependent manner and can attenuate the brain damage^14^, ^15^; however, these inhibitors cannot rescue permanent neuronal damage. The ferrostatins are useful probes which can dissect ferroptosis in a variety of contexts and could form the basis of future drugs to combat lipid‐peroxidation‐mediated tissue injury in many diseases.^16^ Fer‐1, Srs11‐92 (AA9), and their analogs are potent inhibitors of a variety of clinically relevant oxidative cell death phenotypes: AA9 inhibits ferroptosis in erastin‐induced HT‐1080 human fibrosarcoma cells,^16^ and it is efficacious in protecting human and mouse cellular models of Friedreich ataxia with hallmarks of ferroptosis and decreases frataxin deficiency‐induced cell death in healthy human fibroblasts.^17^ Skouta and his colleagues demonstrated that AA9 is 15‐fold more potent than the parent Fer‐1 (with an EC_50_ = 6 nM for erastin lethality suppression), has a high percentage of DPPH inhibition, and is more potent than Fer‐1, and it may be suitable for further translational studies.^17^ However, reports of AA9 in other preclinical studies are limited. In this study, we aim to explain the role of AA9 in neuronal death, nerve injury, and motor dysfunction after ischemic stroke, which may further promote the potential benefits of targeting ferroptosis in stroke, form the basis of future drugs, and bring new clinical treatment options.

## 2 MATERIALS AND METHODS

### 2.1 Animals experiment

Male C57BL/6 N mice (age 8–10 weeks, weight 23–25 g) of specific‐pathogen‐free grade were purchased from the Vital River Laboratory Animal Technology Co., Ltd (Beijing, China). The mice were maintained on a 12/12‐h, light/dark cycle at a temperature (22°C ± 3°C) and humidity (40%–70%) with free access to food and water. All experimental procedures were approved by the Institutional Animal Care Committee of the Xi'an Peihua University, China, in accordance with the National Institutes of Health guidelines. Double‐blind randomization was performed, and the mice were divided into seven groups by the random number method: Sham (*n* = 14), Vehicle (*n* = 14), MCAO (*n* = 19), MCAO + Fer‐1 (*n* = 14), MCAO + AA9 (*n* = 19), MCAO + ML385 (*n* = 19), and MCAO + ML385 + AA9 (*n* = 19) groups. Pentobarbital sodium (40 mg/kg; intraperitoneal injection; Aladdin Reagent Inc., Shanghai, China) was used as an anesthetic, and all groups apart from the Sham group received the MCAO and the blood flow was restored 2 h later. A thermostatic heating pad with 37°C was used during and after surgery to maintain the normal temperature of mice. According to the previous studies,^18^, ^19^ Fer‐1 (Cat: S7243), AA9 (Cat: S9839), and ML385 (Cat: S8790) (Selleck, Houston, USA) were dissolved in 2% DMSO in normal saline and administered intraperitoneally immediately after MCAO (Fer‐1, 2 mg/kg body weight; AA9, 2 mg/kg body weight; ML385, 30 mg/kg body weight). The vehicle group was treated with 20 μL equivoluminal DMSO (2% in normal saline).

### 2.2 Behavioral test

All mice were conducted neurologically tested twice before surgery and 24 h and 72 h after MCAO. The neurological scores were performed by the same researcher, who did not know how the groups were assigned. The neurological deficit was graded on a score from 0 to 5: score 0, no observable neurological deficits; score 1, failed to extend left forepaw; score 2, circled to the left; score 3, fell to the left; score 4, could not walk spontaneously; and score 5, dead. Garcia score assesses voluntary movement in the cage for 5 minutes and includes symmetry of limb movement, symmetry of forelimb extension during tail pulling, climbing the cage wall, response to touching the trunk, and response to touching the whiskers. The maximum score is 18. The lower the Garcia score, the worse the neurological function. The survival mice were also recorded.

### 2.3 Perl's prussian blue staining

After being fixed with 4% paraformaldehyde, dehydrated, and embedded in paraffin, the brain tissue sections were stained with Prussian Blue Iron Stain Kit (Enhance With DAB) (Cat: G1428; Solarbio, Beijing, China) to measure the iron accumulation, according to the manufacturer's instructions. The images were obtained with a light microscope (Olympus, Osaka, Japan).

### 2.4 Iron concentration determination

Brain tissue or primary neurons were obtained and homogenized under 4°C. Iron levels were measured using the tissue iron assay kit (Cat: A039‐2‐1; Nanjing Jiancheng Bioengineering Institute, Nanjing, China), according to the manufacturer's instructions.

### 2.5 Oxidative stress and inflammation analysis

The brain tissues, primary neurons, and culture medium supernatant were collected. A bicinchoninic acid (BCA) assay kit (Cat: 23225; Thermo Scientific) was used to measure the protein concentration. ELISA analysis was conducted to detect oxidative stress (ROS, MDA, SOD, GSH/GSSG, and GPx) and inflammation (TNF‐α, IL‐1β, and HMGB1) by the ELISA Kit, following the manufacturer's instructions (Beyotime, Shanghai, China).

### 2.6 Histological analysis

For Nissl staining, the 4 μm sections were hydrated with 1% toluidine blue (Solarbio, Beijing, China) for 20 min at 50°C. After washing with double distilled water, the sections were dehydrated and mounted. For TUNEL assay, a DeadEnd™ fluorometric TUNEL system (Cat: G3250; Promega, Madison, WI, USA) was used according to the manufacturer's directions, followed by incubation overnight with primary antibodies anti‐NeuN (Cat: ab177487; Abcam, 1:400). The nucleus was stained with 4′,6‐diamidino‐2‐phenylindole (DAPI; Solarbio, Beijing, China), and the image was using a fluorescence microscope (Leica, Oskar‐Barnack, Germany). The stained slices were measured using Image‐Pro Plus (7.0 version).

### 2.7 Immunofluorescence assays

Samples were obtained and fixed with 4% paraformaldehyde, blocked with 5% BSA, and then incubated overnight with primary antibodies: Nrf2 (Cat: 12721; CST, 1:1000) and HMGB1 (Cat: 3935; CST, 1:1000); then, they were incubated with secondary antibodies, the nucleus was stained with DAPI, and the images were photographed using a fluorescence microscope (Leica).

### 2.8 Primary neurons culture

Briefly, hippocampus from day 18 embryonic C57BL/6N mice were used for culture. After removal of the meninges, the cortices were digested in papayotin, and cells were centrifuged at 1000 × *g* for 3 min; neurons were cultured on poly‐L‐lysine‐coated dishes with a density of 1 × 10^6^ cells per 10 cm dish in a minimum of essential medium with 10% horse serum. The medium was changed to neuronal base medium (including 2% B27, 0.2 mM l‐glutamine, and 1% penicillin–streptomycin) on the following day. The medium was changed every 3 days. The cells were cultured for 10 days and then oxygen–glucose deprivation/reperfusion (OGD/R) treatment was conducted. Briefly, the cells were incubated in DMEM and cultured in an incubator containing 95% N2/5% CO_2_ for 4 h. Then, the DMEM was replaced with a standard neuronal culture medium for 24 h. The conventional culture with 5% CO_2_ served as the control. After OGD treatment, cells were immediately treated with Fer‐1 (1 μM), AA9 (1 μM), and ML385 (1 μM) for 24 h, or not. The OGD group received an equal volume of vehicles.

### 2.9 Cell viability analysis

Cell damage was measured by the CCK8 assay. After treatment, 10 μL CCK8 solution (Dojindo Laboratories, Tokyo, Japan) was added to each well of 96‐well plates, incubated at 37°C for 4 h, and measured OD450 with a microplate reader (Thermo Scientific).

### 2.10 Western blot analysis

The sample was lysed with lysis buffer (Beyotime). After measuring the protein concentration (BCA assay kit), electrophoresis was done and the proteins were transferred to the membranes: further, the membranes were blocked with 5% BSA and incubated with the appropriate primary antibodies, Nrf2 (Cat: 12721; CST, 1:1000), HO‐1 (Cat: 86806; CST, 1:800), GPx4 (Cat: ab125066; Abcam, 1:1000), HMGB1 (Cat: 3935; CST, 1:1000), NF‐κB p65 (Cat: 8242; CST, 1:1000), and β‐actin (Cat: 4970; CST, 1:1000) at 4°C overnight. Then, the membranes were further incubated with secondary antibodies (Abgent, 1: 20,000). The signal was detected with an ECL‐Detection Kit (Millipore, USA). The experiments were repeated at thrice in each case.

### 2.11 qRT‐PCR analysis

Total RNA was lysed with Trizol reagent (Invitrogen) and was reverse transcripted to generate cDNA using a HiFi‐MMLV cDNA First Strand Synthesis Kit (CW‐Bio, Beijing, China). qRT‐PCR analysis was executed using GoTaq qPCR Master Mix (Tiangen, Beijing, China). The relative quantification 2^−ΔΔCt^ method was conducted to calculate the mRNA level. GAPDH was used as an internal control.

GPx4, CTGCTCTTCCAGAGGTCCTG (forward: 5′‐3′), GAGGTGTCCACCAGAGAAGC (reverse: 5′‐3′); Nrf2, TCTTGGAGTAAGTCGAGAAGTGT (forward: 5′‐3′), GTTGAAACTGAGCGAAAAAGGC (reverse: 5′‐3′); GAPDH, AAGACCCAGAAATGAAC (forward: 5′‐3′), TCTACACGATAACAACCA (reverse: 5′‐3′).

### 2.12 Statistical analysis

Data are shown as mean ± standard error of the mean (SEM). SPSS 21.0 (IBM, Armonk, NY, USA) software was used to perform data analysis. The Shapiro–Wilk test was used to test the normality. To compare differences between two groups, normally distributed continuous variables were compared by Student's *t* test, while non‐normally distributed variables were compared by the Mann–Whitney *U*‐test. For multiple comparisons of more than two groups, data were analyzed using one‐way analysis of variance (ANOVA) followed by Bonferroni's post‐hoc test with normally distributed or by the Kruskal–Wallis test with non‐normally distributed. The behavioral test was analyzed with repeated measures and two‐way ANOVA, and then Tukey's post‐hoc test was conducted to compare the differences between groups on the same time. *p* < 0.05 was considered significant.

## 3 RESULTS

### 3.1 AA9 inhibited ferroptosis and improved motor behavior in mice after MCAO

**FIGURE 1:**
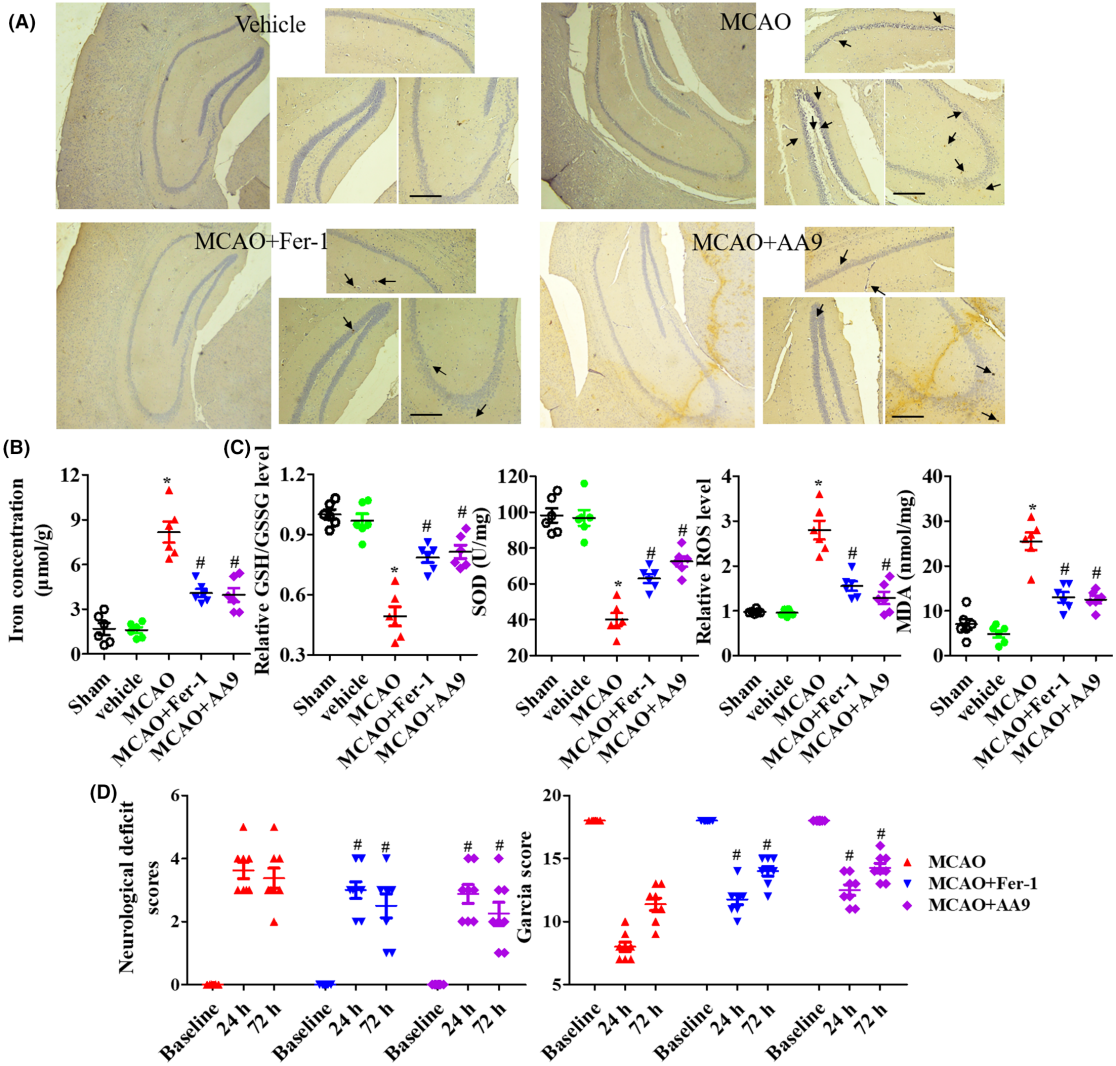
AA9 decreases iron accumulation and improves motor behavior after MCAO. (A) At 24 h after MCAO, Perl's Prussian blue staining was performed to measure the iron deposition in injured brain tissues (black arrow indicated the iron deposition). (B) The iron concentration in the brain tissues on 1 day after MCAO. (C) Measurement of GSH/GSSG ratio, SOD level, ROS accumulation, and MDA level. (D) Fer‐1 and AA9 were administered through intraperitoneal injection immediately after MCAO, and all mice were conducted twice neurological test before surgery and 24 and 72 h after MCAO, including neurological deficit score and Garcia score. Data are presented as the mean ± SEM, *n* = 6. *p < 0.05, vs. sham group; #*p* < 0.05, vs. MCAO group. Scale bar: 50 μm.

IRI caused iron accumulation in the epicenter of injured tissues and iron concentration in brain tissues (Figure 1A, B), and it also significantly induced oxidative stress, including the decrease of GSH/GSSG and SOD levels and increase of ROS and MDA levels (Figure 1C). Both Fer‐1 and AA9 treatment significantly decreased iron accumulation in the epicenter of injured tissues and iron concentration in brain tissues, and improved oxidative stress in brain tissues (*p* < 0.05; Figure 1A–C). All mice were neurologically tested twice before surgery and 24 h and 72 h after MCAO, which presented the same baseline. Both Fer‐1 and AA9 treatment significantly inhibited neurological deficit score and improved Garcia score (Figure 1D), which meant the improvement of motor function.

### 3.2 AA9 enhanced GPx4 level and Nrf2 expression and inhibited inflammation response after MCAO

**FIGURE 2:**
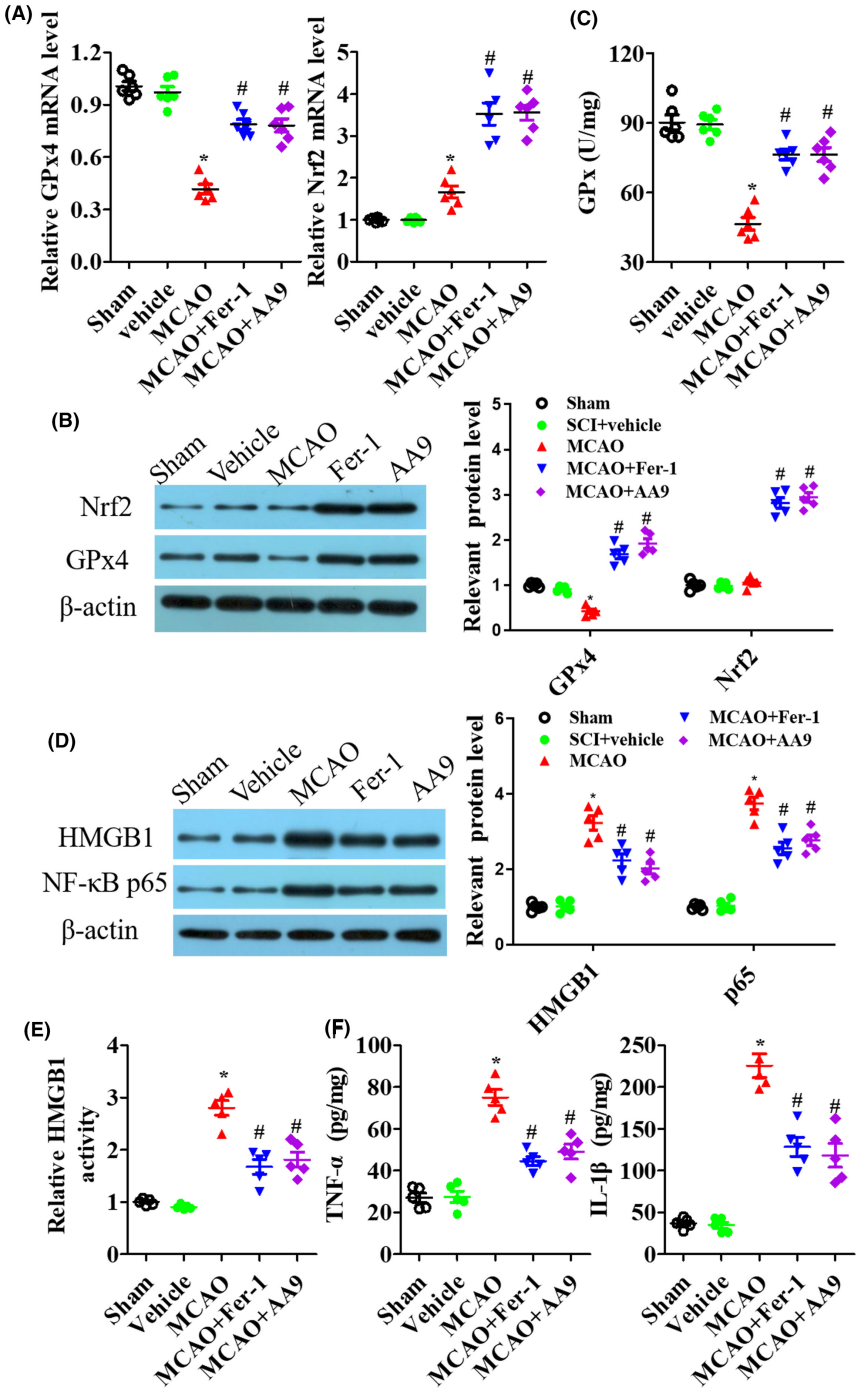
AA9 enhances GPx4 level and Nrf2 expression and inhibits inflammation response after MCAO. At 24 h after MCAO, qRT‐PCR analysis (A) and western blot analysis (B) of the key molecules of ferroptosis GPx4 and Nrf2 levels in the epicenter of injured tissues. (C) GPx activity in the epicenter of injured tissues at 24 h after MCAO. (D) Representative western blot results of HMGB1 and NF‐κB p65 expression in the epicenter of injured brain tissues at 24 h after MCAO, and quantitative analysis of protein expression. ELISA analysis of HMGB1 activation (E) and pro‐inflammatory factors TNF‐α and IL‐1β levels (F) in the epicenter of injured brain tissues at 24 h after MCAO. Data are presented as the mean ± SEM (*n* = 5). *p < 0.05, vs. sham group; #*p* < 0.05, vs. MCAO group.

Then, the key molecules of ferroptosis GPx4 and Nrf2 were measured at 24 h after MCAO. GPx4 mRNA and protein expression were significantly decreased after MCAO, but Fer‐1 or AA9 treatment increased GPx4 level and Fer‐1 and AA9 reversed IRI‐induced GPx activity (Figure 2A–C). For Nrf2 level, IRI significantly induced Nrf2 mRNA level but not protein expression, while Fer‐1 or AA9 greatly promoted Nrf2 mRNA level and protein expression in the epicenter of injured tissues of mice at 24 h after MCAO (Figure 2A, B). As shown in Figure 2C, IRI increased HMGB1 and NF‐κB p65 expression, but Fer‐1 and AA9 significantly suppressed HMGB1 and NF‐κB p65 levels in injured brain tissues after MCAO (*p* < 0.05; Figure 2D). Furthermore, we detected HMGB1 activity and INF‐α and IL‐1β levels by the ELISA assay. Unsurprisingly, HMGB1 activation and pro‐inflammatory factors levels increased rapidly in injured brain tissues, and both Fer‐1 and AA9 significantly cracked down on this phenomenon (*p* < 0.05; Figure 2E, F).

### 3.3 Nrf2 as a key regulator of AA9 reversed IRI‐induced motor dysfunction and ferroptosis in brain

**FIGURE 3:**
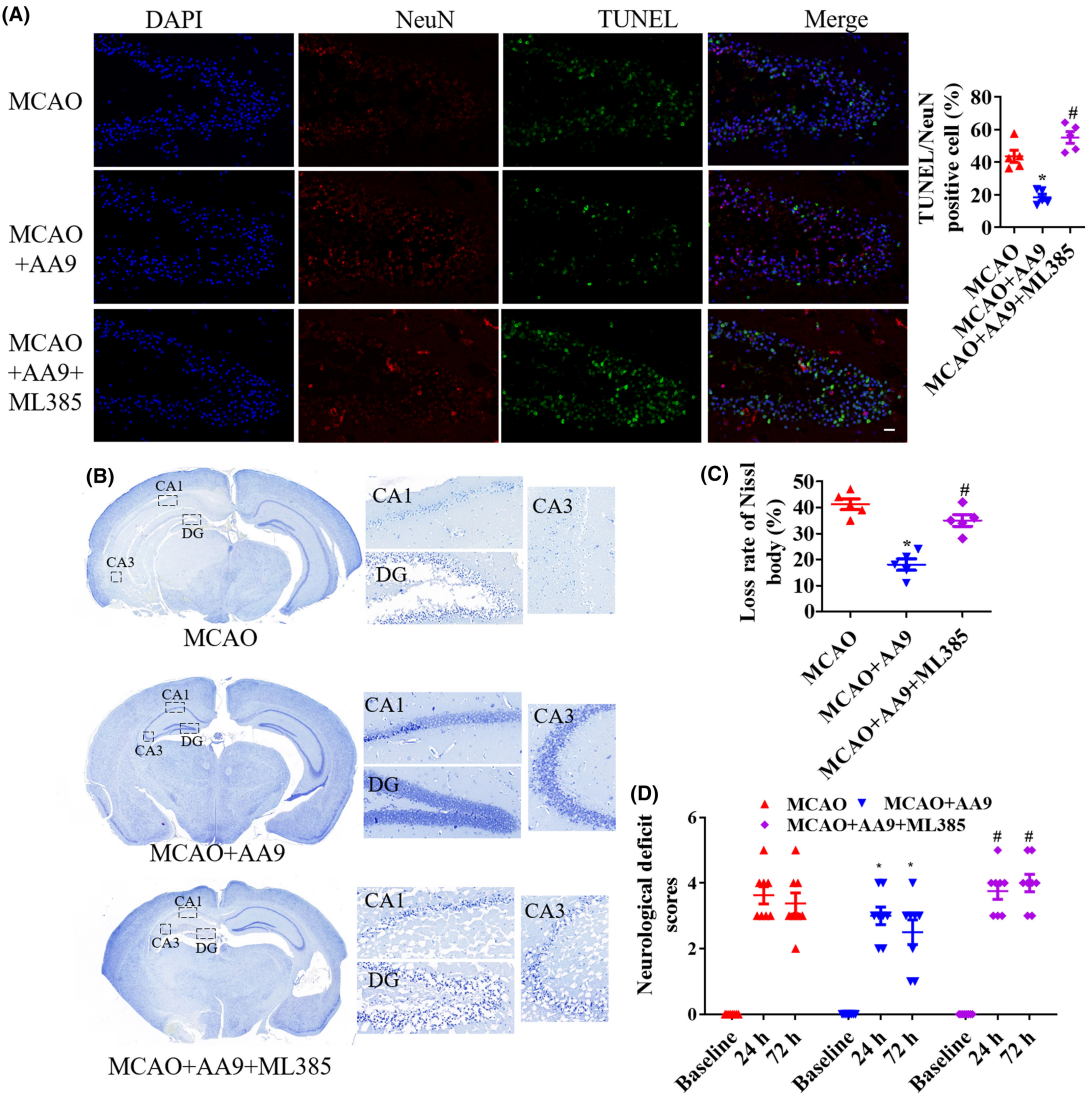
Nrf2 inhibition reduces the neuroprotection of AA9 after MCAO. (A) TUNEL/NeuN staining analysis of injured hippocampal formation at 24 h after MCAO. Dead cells labeled with TUNEL in dentate gyrls with green fluorescence. Nuclei were stained with DAPI and neurons were stained with NeuN. Scale bar: 20 μm. (B) At 24 h after MCAO, Perl's Prussian blue staining was performed to measure the iron deposition in injured brain tissues. (C) Nissl staining analysis of the neuronal damage at 24 h after MCAO. (D) The neurological deficit score analysis of motor behavior at 24 and 72 h after MCAO. Data are presented as the mean ± SEM (*n* = 5). *p < 0.05, vs. MCAO group; #*p* < 0.05, vs. MCAO + AA9 group.

Dead cells labeled with TUNEL in dentate gyrls increased after MCAO but were significantly suppressed following AA9 treatment. ML385 aggravated the neuronal death after MCAO and that eroded the improvement of AA9 in hippocampal formation after MCAO (*p* < 0.05; Figure 3A). Nissl staining showed that Nissl body loss was an important event after MCAO and AA9 treatment improve Nissl body loss, but there was more neuronal damage in the MCAO + ML385 group and Nrf2 inhibition wasted the efforts of AA9 on neuronal protection (Figure 3B, C). Mechanically, ML385 was not friendly to recovery of motor function by Nrf2 inhibition after MCAO, which significantly aggravated motor dysfunction and hindered AA9 protection (*p* < 0.05; Figure 3D).

**FIGURE 4:**
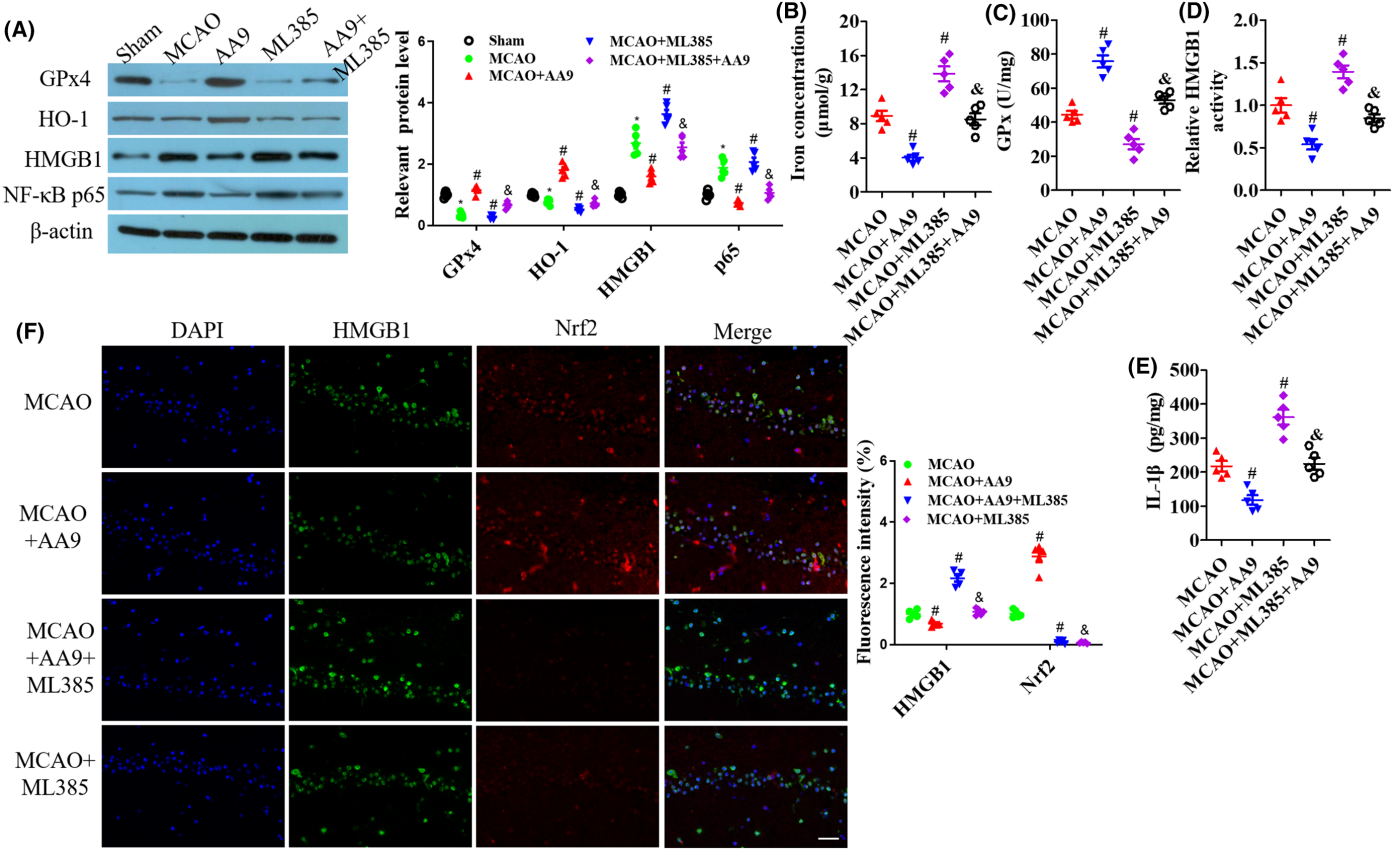
Nrf2 as a regulator of AA9 reverses GPx4 activity and neuroinflammation after MCAO. (A) respective western blot results of protein expression in the epicenter of injured brain tissues at 24 h after MCAO, and quantitative analysis of protein expression. (B) The iron concentration in the brain tissues on 1 day after MCAO. GPx activity(C), HMGB1activation (D), and IL‐1β level (E) in the epicenter of injured tissues at 24 h after MCAO. (F) Nrf2/HMGB1 co‐staining analysis of protein expression at 24 h after MCAO. Nuclei were stained with DAPI. Scale bar: 20 μm. Data are presented as the mean ± SEM (*n* = 5). *p < 0.05, vs. Sham group; #*p* < 0.05, vs. MCAO group; &*p* < 0.05, vs. MCAO + AA9 group.

ML385 suppressed GPx4 and HO‐1 expression and increased HMGB1 and NF‐κB p65 expression in the epicenter of injured tissues at 24 h after MCAO, and it reversed the improvement of GPx4, HO‐1, HMGB1, and NF‐κB p65expression after AA9 treatment (*p* < 0.05; Figure 4A). MCAO + ML385 significantly increased iron concentration (*p* < 0.05; Figure 4B) and inhibited GPx4 activity (*p* < 0.05; Figure 4C), and upregulated HMGB1 activity (*p* < 0.05; Figure 4D) and IL‐1β level (*p* < 0.05; Figure 4E), compared to the MCAO group; and MCAO+ML385 + AA9 presented high iron concentration, low GPx4 activity, and high HMGB1 activity and IL‐1β level (*p* < 0.05; Figure 4B–E), compared to the MCAO+AA9 group. HMGB1 expression was negatively correlated with the Nrf2 level: AA9 upregulated Nrf2 expression and decreased HMGB1level but ML385 weakened AA9‐induced Nrf2 expression after MCAO (Figure 4A, F).

### 3.4 AA9 relieved OGD‐induced neuronal oxidative stress and inflammation via Nrf2 pathway in vitro

**FIGURE 5:**
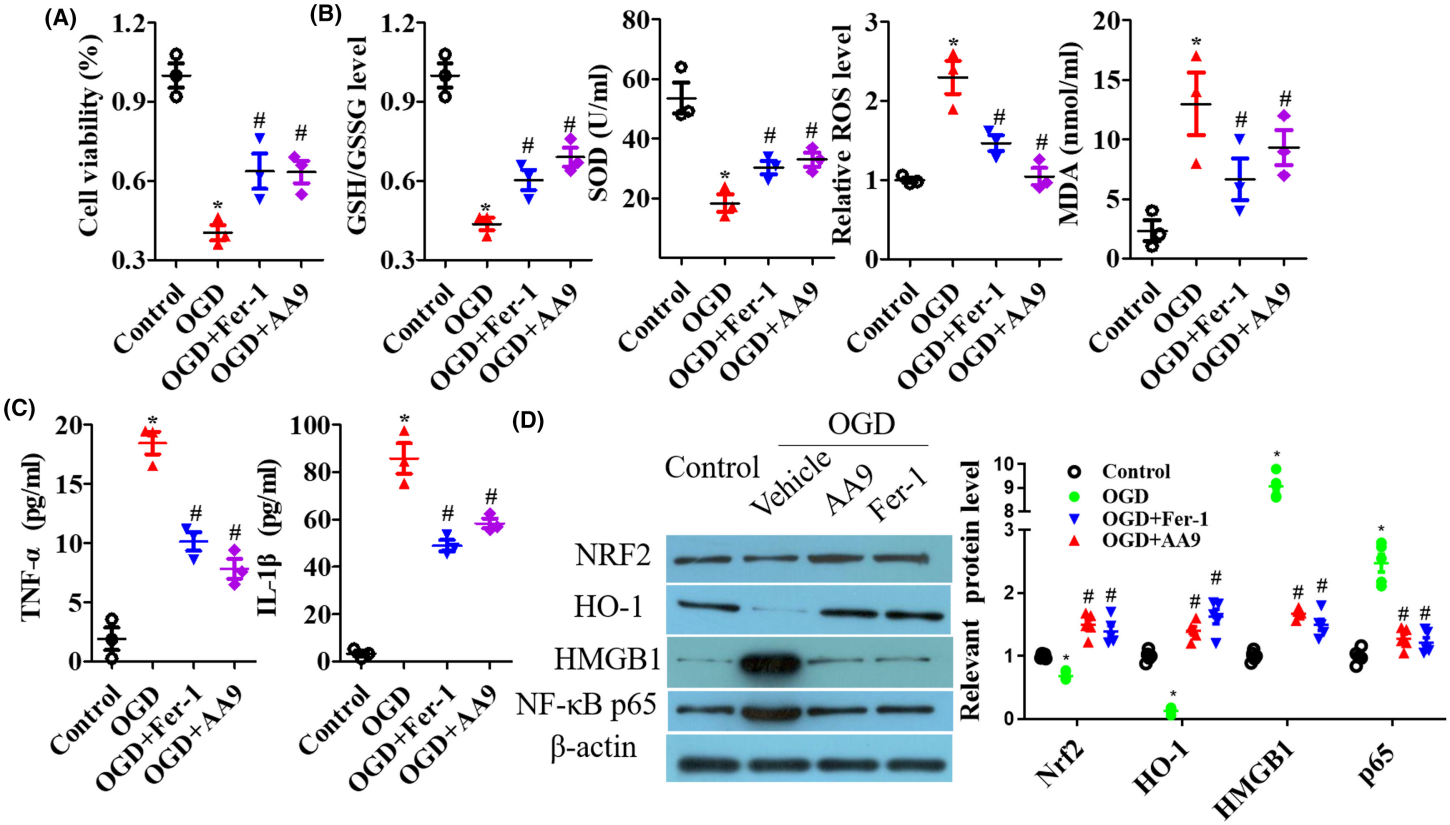
AA9 improves OGD‐induced oxidative stress and inflammation in primary neurons. (A) CCK8 analysis of cell viability. (B) The oxidative stress of GSH/GSSG ratio, SOD level, ROS accumulation, and MDA level in cells were detected. (C) ELISA analysis of pro‐inflammatory factors TNF‐α and IL‐1β levels in cell culture medium supernatant. (D) Western blot results of Nrf2, HO‐1, HMGB1, and NF‐κB p65 expression, and quantitative analysis of protein expression. Data are presented as the mean ± SEM (*n* = 3). *p < 0.05, vs. control group; #*p* < 0.05, vs. OGD group.

**FIGURE 6:**
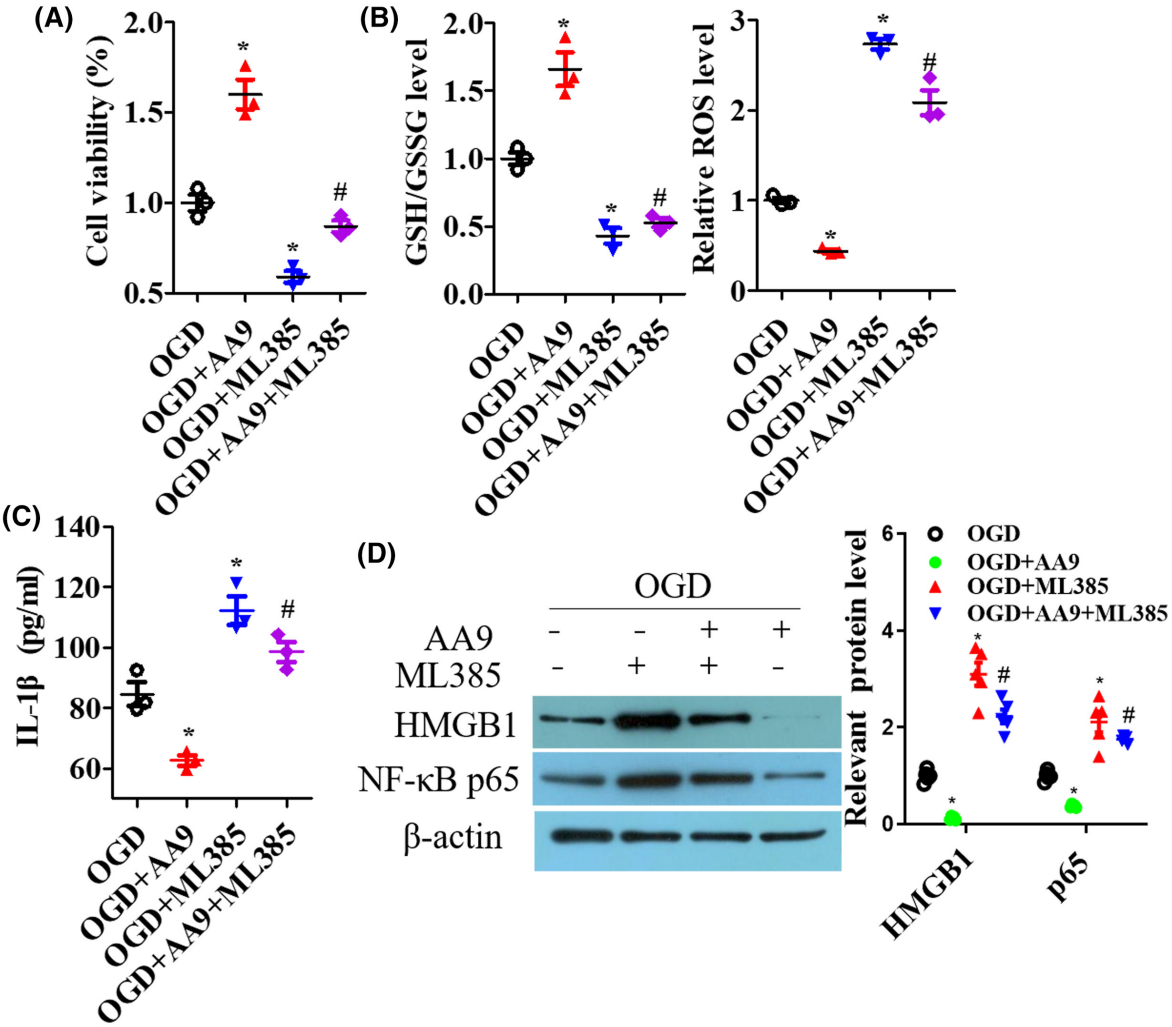
ML385 exacerbates OGD‐induced oxidative stress and inflammation in primary neurons. After OGD treatment, cells were immediately treated with AA9 (1 μM), ML385 (1 μM), or AA9 + ML385 for 24 h, or not. (A) CCK8 analysis of cell viability. (B) The oxidative stress of GSH/GSSG ratio and ROS accumulation in cells were detected. (C) ELISA analysis of pro‐inflammatory factor IL‐1β levels in cell culture medium supernatant. (D) Western blot results of HMGB1 and NF‐κB p65 expression, and quantitative analysis of protein expression. Data are presented as the mean ± SEM (*n* = 3). *p < 0.05, vs. OGD group; #*p* < 0.05, vs. AA9 group.

To investigate the neuroprotection of AA9, primal neurons were immediately treated with 1 μM Fer‐1 or AA9 for 24 h, or not, after OGD, then oxidative stress and inflammation were detected. Neuronal viability was suppressed by OGD and Fer‐1 and AA9 significantly reversed the impairment of cell viability (*p* < 0.05; Figure 5A). For oxidative stress and inflammation detection, OGD enhanced ROS, MDA, TNF‐α, and IL‐1β levels and subdued GSH/GSSG ratio and SOD level in neurons but Fer‐1 and AA9 significantly improved the levels of these molecules (*p* < 0.05; Figure 5B, C). Nrf2, HO‐1, HMGB1, and NF‐κB p65 expressions were measured; Fer‐1 and AA9 also upregulated Nrf2 and HO‐1 expression, and downregulated HMGB1 and NF‐κB p65 expression to resist the damage by OGD in primal neurons (*p* < 0.05; Figure 5D). Furthermore, Nrf2 inhibitor ML385 exacerbated the impairment of neuronal viability (*p* < 0.05; Figure 6A), oxidative stress (*p* < 0.05; Figure 6B), pro‐inflammatory factors levels (*p* < 0.05; Figure 6C), and the expression of HMGB1 and NF‐κB p65 (*p* < 0.05; Figure 6D) in the neuron OGD model, and that eroded AA9‐improved neuronal oxidative stress and inflammation via the inhibition of expression of Nrf2 (Figure 6A–D).

## 4 DISCUSSION

Stroke causes severe disability and mobility problems worldwide, and due to the lack of treatment, the development of viable new drugs is urgent for stroke. In this work, we demonstrated that ferroptosis inhibitor Fer‐1 and its analog AA9 improved brain infarct size and neurological deficits in a mouse model of MCAO, and inhibits ischemic stroke‐induced oxidative stress and inflammation in the epicenter of injured brain tissues in damaged hippocampus. However, Nrf2 inhibitor ML385 reversed the neuroprotection of AA9. In vitro, AA9 relieved OGD‐induced neuronal oxidative stress and inflammation via Nrf2 pathway, which was impaired by ML385 in primary neurons.

Ferroptotic pathological phenomena play an important role in cerebral IRI injury.^20^ Some studies have evaluated the safety and explored the therapeutic efficacy of the iron chelator and antioxidant DFO or NAC in ischemic stroke patients.^21^, ^22^ Among patients with moderate hematoma volume (HV) after ICH, a greater proportion of DFO‐ than placebo‐treated patients achieve a modified Rankin Scale score of 0–2 but no significant function on small or large HVs.^22^ Oral NAC early after acute ischemic stroke has a better prognosis for acute neurological deficits and disability, partly because of its antioxidant and anti‐inflammation.^21^ In addition, selenium supplementation can effectively inhibit GPx4‐dependent iron death and endoplasmic reticulum stress‐induced cell death by promoting GPx4 expression after ischemic stroke.^23^ Some Chinese herbs containing flavonoids and phenolic acids participate in the neuroprotective effect of anti‐oxidation and anti‐apoptosis by regulating ferroptosis and alleviating cerebral IRI in vivo and in vitro.^24^ These studies suggest that reversing ferroptosis after IRI may be a possible event for improving neuronal injury.

Fer‐1 inhibits oxidative lipid damage and cell death in diverse disease models. Fer‐1 alleviates cardiac dysfunction and inflammation in myocardium of septic rats via TLR4/NF‐κB signaling.^25^ Fer‐1 restores synaptic protein expression by inhibiting the activation of P38 MAPK, thereby alleviating cognitive dysfunction in epileptic rat.^26^ Intraventricular injection of Fer‐1 reduces iron deposition and neuronal degeneration, alleviates injury lesions, and improves long‐term motor and cognitive function.^27^ Reports have shown that ICH‐induced mice neurological deficits, memory impairment, and brain atrophy are reduced by Fer‐1 treatment,^28^ and the administration of Fer‐1 upregulates Fpn, decreases the iron content, and then improves the lipid peroxidation and early brain injury in subarachnoid hemorrhage rats.^29^ However, the reports of AA9 lacking, AA9 protects the healthy medium spiny neurons in rat corticostriatal brain slices from huntingtin‐induced cell death and oligodendrocytes from cystine deprivation and reduces human and mouse cellular models of Friedreich ataxia with hallmarks of ferroptosis.^16^, ^17^ Skouta and his colleagues discover that AA9 is more potent than Fer‐1 and may be suitable for further translational studies.^16^ We discussed the possible role of AA9 in ischemic stroke and demonstrated that AA9 and Fer‐1 protected neurological deficits and neuronal ferroptosis. Nrf2 is a major transcription factor that regulates cellular resistance to endogenous and exogenous stress and is a promising therapeutic target for stroke.^30^, ^31^ Furthermore, Nrf2 promotes the transcription of HO‐1 genes related to antioxidant reaction^32^ and upregulates Nrf2/HO‐1 that decreases neuron damage and neurological dysfunction in MCAO and ICH animal models.^33^, ^34^

Inadequate activation of Nrf2 in humans has been associated with chronic diseases such as Parkinson's disease, Alzheimer's disease, and multiple sclerosis, and acute oxidative stress to the brain, such as stroke and traumatic brain injury, is high in animals that are deficient in Nrf2.^35^, ^36^ The new findings also link activation of the Nrf2 pathway with NF‐κB interactions and anti‐inflammation.^36^, ^37^, ^38^, ^39^, ^40^ Kobayashi et al. demonstrated that Nrf2 interferes with lipopolysaccharide‐induced transcriptional upregulation of proinflammatory cytokines IL‐6 and IL‐1β, and Nrf2‐mediated inhibition is independent of the Nrf2‐binding motif and ROS level.^38^ Fu et al. showed that isoliquiritigenin attenuates Aβ42‐induced inflammation and oxidative stress in BV2 cells via the regulation of Nrf2/NF‐κB signaling.^41^ Phloretin reduces the inflammatory phenotype of macrophages and markedly suppresses neuroinflammation in experimental autoimmune encephalomyelitis by Nrf2 activation in macrophages.^37^ Dimethyl fumarate improves cognitive impairment and reverses hippocampal neuron damage and loss in rats with chronic cerebral hypoperfusion by inhibiting hippocampal neuroinflammation, oxidative stress, and ferroptosis via Nrf2/ARE/NF‐κB signal pathway.^36^ In this work, we found that AA9 suppressed HMGB1 and NF‐κB p65 expression in the epicenter of injured brain tissues in damaged hippocampus. However, Nrf2 inhibitor ML385 reversed the neuroprotection of AA9, including oxidative stress and neuroinflammation. In vitro studies show that AA9 relieved OGD‐induced neuronal inflammation via Nrf2 pathway, which was impaired by ML385 in primary neurons. Hydrogen suppresses neurobehavioral deficits, apoptosis, inflammation, and oxidative stress via the Nrf2‐mediated NLRP3 and NF‐κB pathways but the Nrf2 deficiency abolishes the inhibited effect of hydrogen on the expression of NLRP3 pathway members and p65 NF‐κB after hypoxic–ischemic injury (HII).^39^ Chlorogenic acid presents neuroprotection against HII in neonatal rats by regulating the Nrf2/NF‐κB signaling pathway.^40^ Therefore, the AA9 activates the bi‐directional switch Nrf2 to inhibit oxidative stress and neuroinflammation, and the inflammatory response is partly dependent on the Nrf2 Nrf2/NF‐κB signaling pathway.

## 5 CONCLUSIONS

In conclusion, our data revealed that AA9 improved brain infarct size and neurological deficits in the mice model of MCAO, and it also inhibits ischemic stroke‐induced neuronal damage, by rescuing iron deposition, ROS accumulation, and GPX4, Nrf2, HO‐1, HMGB1, and NF‐κB p65 expression. The findings imply that Fer‐1 analog AA9 may be suitable for further translational studies for the protection of neurological deficits in stroke via Nrf2 signal pathway‐mediated neuronal damage, including overloaded oxidative stress and inflammation. The data presented in this study support the idea of further clinical trials of targeted therapies for ferroptosis in stroke. In addition, given the specificity of AA9 for ferroptosis, another implication of these data is that ferroptosis inhibitors may have therapeutic potentiality and could form the basis of future drugs to combat lipid‐peroxidation‐mediated neurological diseases.

## CONFLICT OF INTEREST STATEMENT

The authors declare that they have no competing interests.

## Supporting information


Appendix S1

## Data Availability

The data that support the findings of this study are available from the corresponding author upon request.
