# Supplementary material for: Srs11‐92, a ferrostatin‐1 analog, improves oxidative stress and neuroinflammation via Nrf2 signal following cerebral ischemia/reperfusion injury
Source: CNS Neurosci Ther. 2023 Feb 27;29(6):1667–77. doi: 10.1111/cns.14130 (PMC10173707; doi:10.1111/cns.14130)

Full unedited gel/blot for Figure 2B and 2D

Nrf2

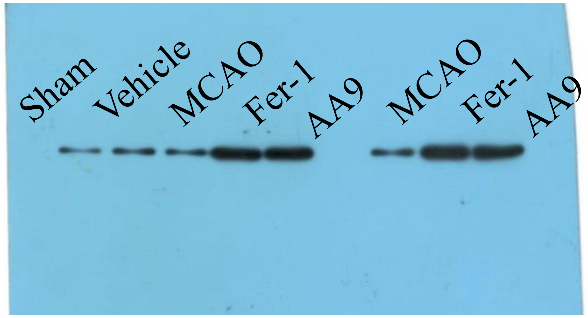

HMGB1

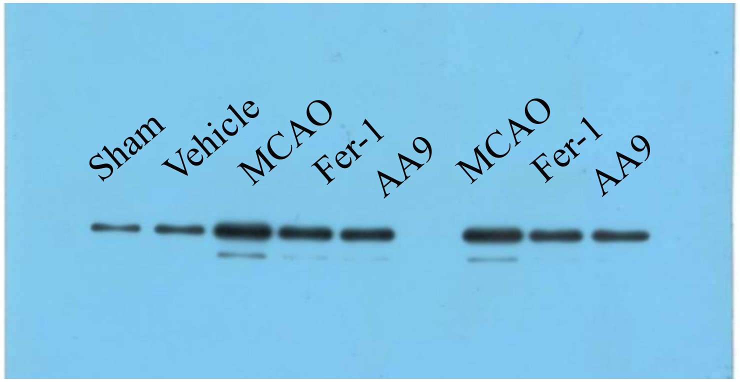

GPx4

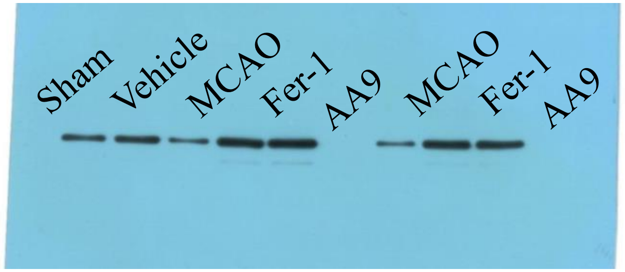

NF-κB p65

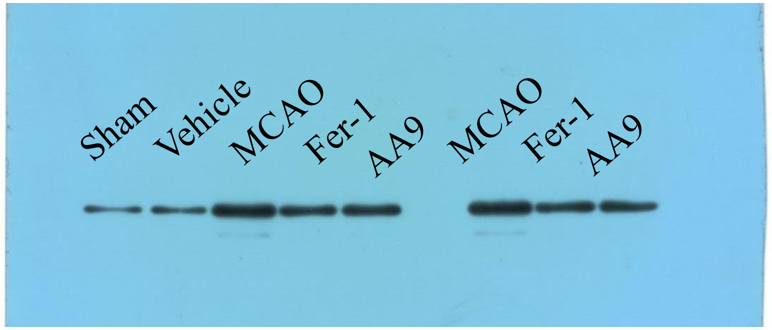

β-actin

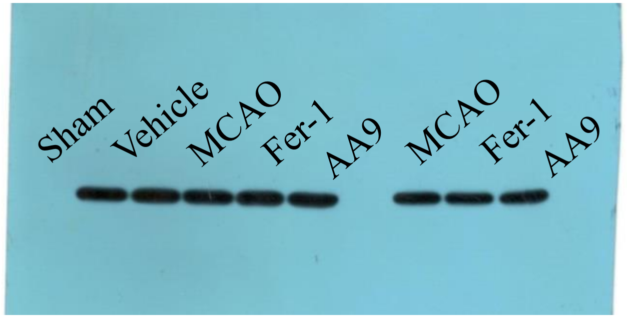

β-actin

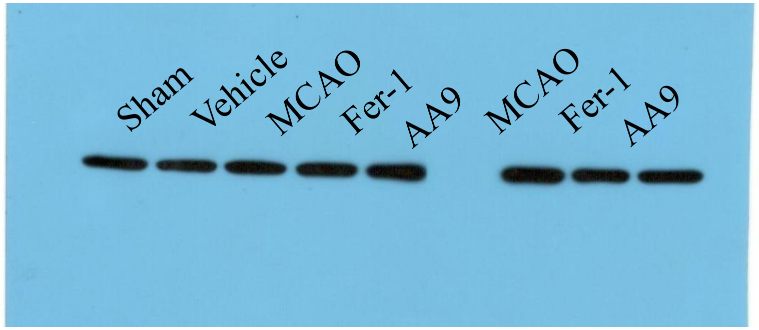

Full unedited gel/blot for Figure 4A

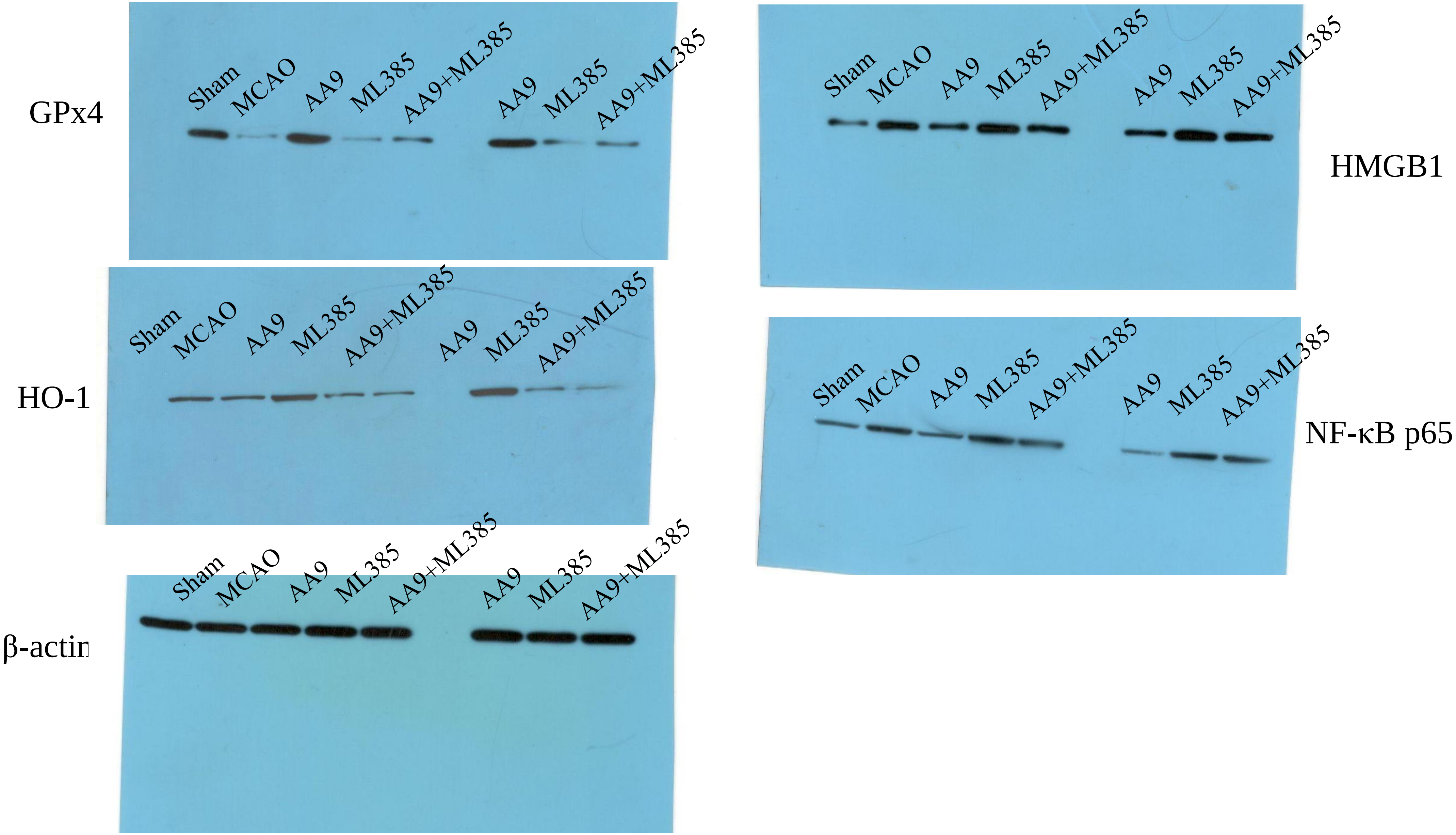

Full unedited gel/blot for Figure 5D and 6D

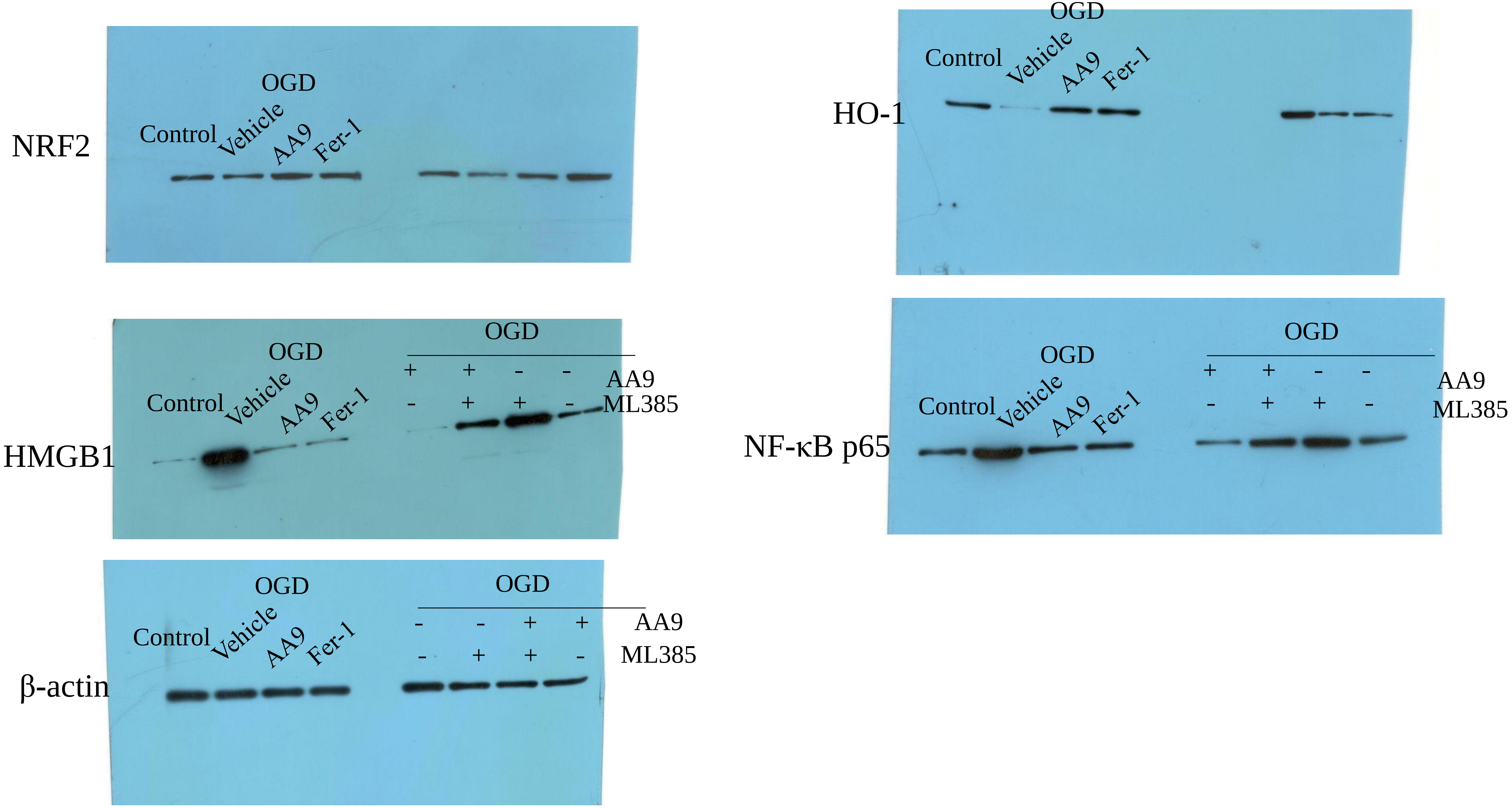

Supplement: Supplementary file 1 — Appendix S1 [file CNS-29-1667-s001.pdf]
